# Exploring bilateral pneumothorax leading to a rare diagnosis in a young lady

**DOI:** 10.1002/rcr2.1180

**Published:** 2023-06-09

**Authors:** Harshana Bandara, Dushantha Madegedara

**Affiliations:** ^1^ Respiratory Medicine Unit National Hospital of Kandy Kandy Sri Lanka

**Keywords:** bilateral pneumothorax, chylous pleural effusion, lung cysts, lymphangioleiomyomatosis

## Abstract

Bilateral pneumothorax is a rare occurrence and vigilant clinical examination is mandatory to suspect that during the presentation. This case illustrates a young lady presented with bilateral pneumothorax, new identification of lung cysts and chylous pleural effusion leading to diagnosis of lymphangioleiomyomatosis.

## Graphical abstract

This case illustrates a young lady presented with bilateral pneumothorax, new identification of lung cysts and chylous pleural effusion leading to the diagnosis of lymphangioleiomyomatosis.

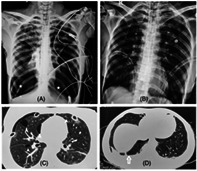

## CLINICAL IMAGE

**FIGURE 1:**
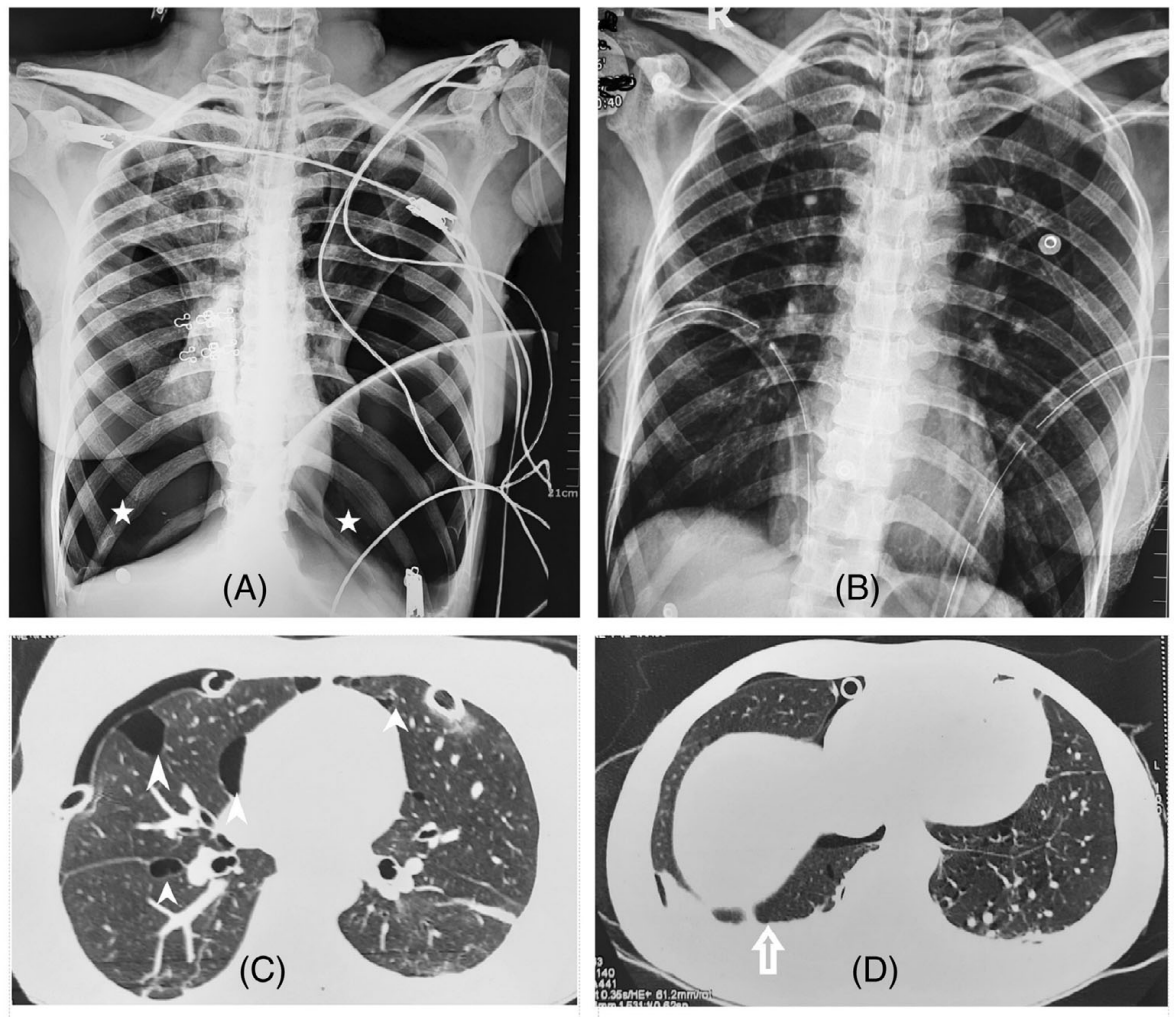
The chest x‐ray showing bilateral pneumothorax in (A) (white asterixis) before the chest drains insertion. The resolution of the pneumothorax following chest drain insertion in (B). The axial views of computed tomographic imaging showing bilateral thin‐walled pulmonary cysts in (C) (white arrowheads) and thin pleural effusion in (D) (white arrow).

A 27‐year‐old lady presented to our hospital with abrupt severe breathlessness with chest tightness. She was sweating, severely tachypnoeic, tachycardic and hypoxic. Chest examination revealed central trachea, bilaterally reduced breath‐sounds and hyperresonance on percussion. Chest sonography suspected bilateral pneumothorax and chest x‐ray (Figure 1A) confirmed bilateral pneumothorax. Bilateral chest drains were inserted (Figure 1B) with rapid symptom relief with stabilization of vitals. The whitish fluid noted on the drain and the samples showed evidence of chylothorax with a triglyceride level of 140 mg/dL.

The high‐resolution computed‐tomography (Figure 1C, D) of chest showed multiple thin‐walled cysts with bilateral pleural effusions and resolution of pneumothorax. The bilateral pneumothorax, chylothorax and bilateral thin‐walled lung cysts of this patient in childbearing age led to the diagnosis of lymphangioleiomyomatosis.

Lymphangioleiomyomatosis is a rare cystic lung disease with involvement of lymphatics.^1^ The commonest group involved are females in child‐bearing age with a mean age of 34‐years.^1^ The commonest features are pneumothorax, progressive breathlessness and chylous‐pleural effusions.^1^, ^2^ Presentation with bilateral pneumothorax itself is an extremely rare presentation and it is definitely a diagnostic challenge due to the symmetrical examination findings with central trachea. Therefore, in the situation of clinical suspicion, elicited by bilateral hyper‐resonant percussion note, emergency ultrasound is crucial in detecting/suspecting bilateral pneumothorax.

## AUTHOR CONTRIBUTIONS

Both authors contributed to the conception of the case report. Harshana Bandara wrote the first draft. Dushantha Madegedara revised subsequent versions and approved the final version.

## CONFLICT OF INTEREST STATEMENT

None declared.

## ETHICS STATEMENT

The authors declare that appropriate written informed consent was obtained for the publication of this manuscript and accompanying images.

## Data Availability

Data sharing not applicable to this article as no datasets were generated or analysed during the current study.
